# Rapid detection of drug-resistant *Mycobacterium tuberculosis* by Modified MODS assay suitable for resource-poor settings

**DOI:** 10.1371/journal.pntd.0011852

**Published:** 2024-01-04

**Authors:** Bashir Ahamd Fomda, Gulnaz Bashir, Sehrish Baqal, Yaawar Bashir Mir, Rehana Ali, Altaf Hussain Khan, Asiya Khan, Anis Bashir, G. M. Chuloo

**Affiliations:** 1 Department of Microbiology, Sher-i-Kashmir Institute of Medical Sciences, Srinagar, Jammu and Kashmir, India; 2 All India Institute of Medical Sciences, New Delhi, India; 3 State TB Officer, Directorate of Health Services, Kashmir Province, Srinagar, Jammu and Kashmir, India; NIAID-ICER, INDIA

## Abstract

**Background:**

Cross contamination and biosafety are concerns with the microscopic observation drug susceptibility assay. To address these issues, we modified the MODS technique in the current study.

**Methodology/Principal findings:**

Two hundred and seventy-five samples were processed on LJ media and drug susceptibility was performed by the Indirect agar proportion method. A modified MODS test was done in tissue culture bottles. GenoType MTBDR*plus* assay was performed to detect the resistance and mutational pattern associated with the resistances. Sensitivity, specificity, positive predictive value, and negative predictive value for the detection of tuberculosis by modified MODS were 97.44%, 80.00%, 97.44%, and 80.00% respectively. The perfect agreement was seen between modified MODS and the Indirect agar proportion method for drug susceptibility testing of isoniazid (kappa = 0.923) and rifampicin (kappa = 1). The contamination rate, cost and TAT for modified MODS were less as compared to the solid media.

In the case of MDR-TB isolates S531L (66.66%) was the most prevalent mutation in the *rpoB* gene followed by S315T2 mutation (58.33%) and T8C (41.66%) in *katG* and *inhA* gene respectively. In hetero-resistant strains, C-15T mutation (37.50%) was the most common followed by A-16G (12.50%) in *the inhA* gene. In INH mono-resistant strains only two mutations were observed i.e., S-315T1(50%) and C-15T (50%) in the *katG* and *inhA* genes respectively.

**Conclusions/Significance:**

Modified MODS proved to be cost-effective and user-friendly, with minimal risk to the handler and no cross-contamination between samples were observed. Hence, it can be used in low-income countries for early detection of tuberculosis and its resistance.

## Author summary

Tuberculosis is a major health problem, particularly in developing countries. Globally efforts are being made to control this disease. However, the emergence of multi-drug resistance (MDR) is a major threat to the tuberculosis control program. Moreover, the available methods for the detection of tuberculosis and susceptibility testing are time-consuming and labour-intensive. All these methods require pre-culture before drug susceptibility testing hence the turnaround time is prolonged. The microscopic observation drug susceptibility method is a liquid-based culture method which simultaneously detects tuberculosis and its susceptibility.

Originally developed microscopic observation drug susceptibility assay is done in 24 well tissue culture plates, but problems of false positivity which likely represent cross-contamination from another positive specimen or positive controls plated on each MODS plate is the limitation of this assay. This method also poses the risk of exposure to infection to the person performing the assay. To overcome these issues, in the present study, we introduced modified MODS using screw-capped tissue culture bottles instead of tissue culture plates. The advantage of this modification is that samples and controls are processed in separate bottles which are screw-caped hence reducing chances of cross-contamination and risk of exposure to infection. The mutational pattern of drug-resistant strains is not known in this region. GenoType MTBDR*plus* assay was performed to detect the resistance and mutational pattern associated with the resistances.

## Introduction

Tuberculosis is a global public health priority being one of the leading causes of death from a single infectious agent after COVID [1]. The emergence of multi-drug resistant (MDR) strains worldwide is a major threat to the tuberculosis control program, and this is compounded by a substantial decrease in drug resistance detection due to the COVID-19 pandemic [2]. Improvement and expansion of affordable tuberculosis diagnostic tests are urgently needed to increase the detection of tuberculosis, multidrug-resistant TB and extensively drug-resistant-TB worldwide [2–5]. Historically sputum smear microscopy was used for the diagnosis of tuberculosis. Although this method is inexpensive, it has a relatively low limit of detection, and it cannot be used for drug susceptibility testing. Rapid molecular methods such as GeneXpert MTB/ RIF (Cepheid, Sunnyvale, CA, USA) and Genotype MTBDR*plus* are available, but the cost per test is too high for low-income countries [6–8]. The currently available methods using solid media are inexpensive but slow and laborious. Liquid automated commercial systems such as the BACTEC MGIT 960 (Becton Dickinson, Sparks, MD), are rapid but require heavy, expensive equipment, have high running costs, and are technically complex [9–12]. All these methods require pre-culture before drug susceptibility testing hence the turnaround time is prolonged. To overcome the need for preculture before drug susceptibility testing, the microscopic observation drug susceptibility (MODS) method has been developed [13–16]. The microscopic observation drug susceptibility assay is an inexpensive, simple, culture-based technique for the diagnosis and detection of drug-resistant tuberculosis [17,18]. In most of the studies, 24 well tissue culture plates have been used for MODS, but problems of false positivity which likely represent cross-contamination from another positive specimen or the H37Rv positive controls plated on each MODS plate have important implications for both patient care and decentralization of assay [19]. Moreover, there is a concern for biosafety. To address these issues, in the present study, we introduced modified MODS, a modification in MODS using screw-capped tissue culture bottles instead of tissue culture plates. We evaluated the accuracy of modified MODS in comparison to the Lowenstein Jensen (LJ) and agar proportion method as the gold standard for drug susceptibility testing of *M*. *tuberculosis*. We aimed to evaluate the time to positivity, cost per sample, ease of performance of each test and to determine the mutations associated with resistance.

## Materials and methods

### Ethics statement

The study was approved by the Institutional Ethical Committee of the Sher-i-Kashmir Institute of Medical Sciences via Protocol No 199/2014. A written consent was obtained from the patients included in the study.

### Study design

This was a cross-sectional, hospital-based study, conducted over 3 years. All new cases who were registered as sputum smear positive, and patients previously treated but continue to be sputum smear-positive, including default & relapse cases (Category II) were included in this study. Patients registered as sputum smear positive and on anti-tubercular treatment (ATT) were excluded from the study.

### Sample size calculation

To ascertain the number of subjects needed for our prospective study, we used the confidence width method. The prevalence rate of MDR TB in India is 11–49% as reported by Sharma et al [12] and 6% has been reported from Kashmir by Bikram et al [13]. The sample size was calculated by taking the mean of lowest prevalence and higher prevalence and achieving a 95% confidence interval length at a 5% confidence limit using the following formula: N = Z^2^Pq/d^2^. The calculated sample size was 235 and we collected 275 sputum samples from smear-positive TB patients.

### Sample collection and processing

Sputum specimens (2 ml) were collected in 25ml sterile wide-mouthed plastic containers and were processed immediately; however, in case of delay, they were kept at 4°C. Samples from the outskirts were transported in an equal quantity of cetylpyridinium chloride (CPC). Sputum samples were directly subjected to decontamination using N-Acetyl-L-cysteine and sodium hydroxide (NALC-NaOH) method [19]. Subsequently, the sediments were suspended in 1–1.5 ml sterile phosphate buffer (pH 6.8). Smear microscopy was done by the Ziehl-Neelsen staining method and smears were graded as per guidelines of the National Tuberculosis Elimination Programme (NTEP). All samples were processed in a class II biosafety cabinet.

### Culture on LJ media

A hundred microliters of suspended sediment were inoculated on LJ medium which was incubated at 37°C and inspected first after three days and then daily for eight weeks. If contamination was present, the cultures were discarded and repeated from the backup samples. All positive cultures were identified by Ziehl-Neelsen staining, their slow growth rate, colony morphology and biochemical tests as M. tuberculosis. All the positive samples were stored in 20% glycerol at -70°C till further use.

### Indirect agar proportion method using Middlebrook 7H10 Agar

The drug susceptibility test was carried out according to the instructions of the Clinical and Laboratory Standards Institute [16]. The drug concentrations used in the medium were 0.2 mg/mL for isoniazid (INH) and 1.0 mg/mL for rifampicin (RIF). The numbers of colonies observed on the drug-containing medium were then compared with the numbers on the drug-free medium. The proportion of bacilli resistant to a given drug was determined and expressed as a percentage of the total population tested. If the ratio was more than 1% the isolate was considered resistant. Resistance to both isoniazid (INH) and rifampicin (RIF) was considered MDR-TB (Multi-drug-resistant tuberculosis).

## Modified MODS

This was done using tissue culture bottles, a modification over the conventional 24-well microtiter plates. This method only required an inverted microscope and tissue culture bottles for the detection of the TB. Middlebrook 7H9 (MB7H9) broth was prepared by using MB7H9 broth base, glycerol (0.31%), oleic acid-albumin-dextrose-catalase (OADC) (10%) containing polymyxin B, amphotericin B, nalidixic acid, trimethoprim, azlocillin (PANTA) antibiotic (Hi-Media Laboratories Ltd, Mumbai, India.) and Bacto casitone (0.125%) (Becton, Dickinson and Company, Sparks, MD).

### Inoculation of bottles

For each sample, three tissue culture bottles were used and labelled as GC (Growth Control), INH and RIF. Each of the tissue culture bottles was then dispensed with 5ml of MB7H9-OADC medium, 0.1ml PANTA and 0.1 ml of the decontaminated sample. To RIF and INH labelled bottles 400μl of RIF and INH working solutions were added to give final concentrations of 1 μg/ml (RIF) and 0.2 μg/ml (INH) respectively. One tissue culture bottle containing only MB7H9-OADC media was used as internal quality control (media control). The tissue culture bottles were immediately closed and incubated at 37°C. These bottles were observed daily under an inverted microscope (EVOS XL CORE with a screen instead of an objective which made the viewing of bottles very easy and less tedious. The date of the first appearance of chording suggestive of mycobacterial growth was noted. A fully susceptible strain of *M*. *tuberculosis* H37Rv (ATCC- 27294) was used as a positive control.

### Interpretation of modified MODS assay

**Fig 1:**
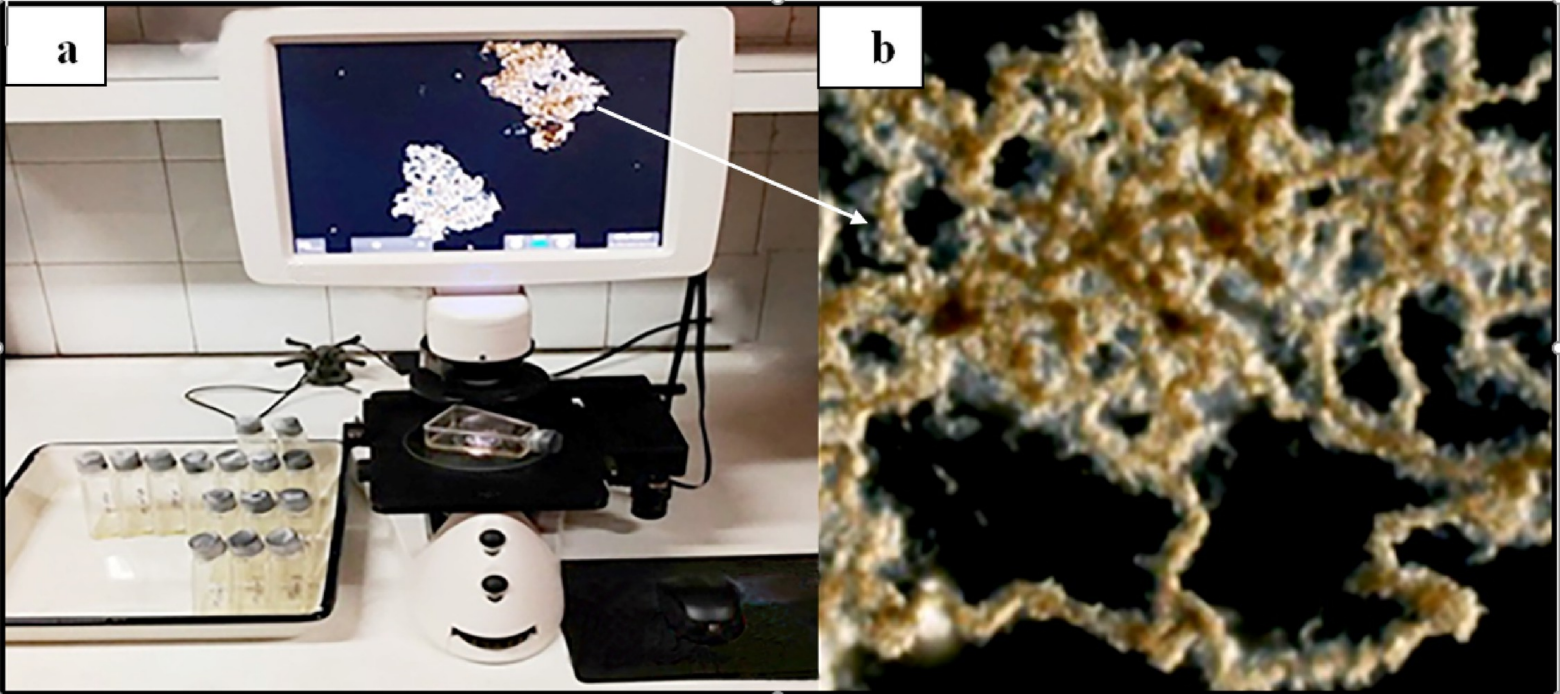
**(a)** *M*. *tuberculosis colonies* as seen under the inverted microscope (x 400). **(b)** Characteristic serpentine cords of *M*. a *tuberculosis* observed under the inverted microscope (x 400).

A strain was considered susceptible if ≥ 2 cord-like structures (≥2 CFU) were observed in the drug-free bottles but not in a drug-containing bottle. A strain was considered resistant if ≥ 2 cord-like structures were observed in both the control and drug-containing bottles. The appearance of 1 CFU in either the drug-free bottle or in both was considered indeterminate. When a positive result was observed (≥ 2 CFU) in the drug-free bottle, the isoniazid and rifampicin-containing bottles were also examined on the same day. Negative bottles were further incubated and read every day. If the result was negative on day 21, the final result was interpreted as negative. (**Fig 1A and 1B**).

### Genotype MTBDR*plus* assay

GenoType MTBDR*plus* assay using GenoType MTBDR*plus* kit (Hain Life science GmbH Nerhen, Germany) was performed and interpreted as per the manufacturer’s instructions (https://www.hain-lifescience.de/en/products/microbiology/mycobacteria/tuberculosis/genotype-mtbdrplus.html).

### Statistical analysis

Data were presented as frequency (percentage) and mean (SD). Diagnostic parameters such as sensitivity, specificity, negative predictive value, and positive predictive value with 95% confidence intervals were calculated using 2x2 contingency tables. Categorical variables were analysed by Chi-square/Fisher’s exact test. We also used Cohen’s kappa to summarise the agreement between different methods. All confidence intervals reported are two-sided 95% confidence intervals, with p-values of 0.05 considered statistically significant. All analyses were performed with Stata version 11.2 (StataCorp, Texas, USA).

## Results

Demographic characteristics and patient profiles are shown in Table 1. A total of 275 patients were enrolled in the study. Out of these 192 (69.8%) were males and 83 (30.2%) were females with male to female ratio of 2.2:1. The Median age was 49 years (IQR 60–30).

**Table 1:** Demographic characteristics and patient profile.

Variables		Number (N)	(%)
**Sex**	**Male**	192	69.8
	**Female**	83	30.2
**Age (Years)**	**0–14**	05	1.82
	**15–30**	71	25.8
	**31–45**	52	18.9
	**46–60**	79	28.7
	**61–75**	55	20
	**76–90**	11	4
	**>91**	2	0.73
**TB History**	**New Case**	261	95
	**Relapse**	10	3.6
	**Defaulter**	4	1.45
**Total**		275	100

Based on National Tuberculosis Elimination Programme (NTEP) guidelines AFB grading of smear-positive specimens was done. Twenty-five (10%) were 3+, 72 (26%) 2+, 161 (58%) 1+, and Seventeen (6%) were scanty. Thus, most specimens were of grade 1+, followed by grade 2+, grade 3+ and scanty.

**Fig 2:**
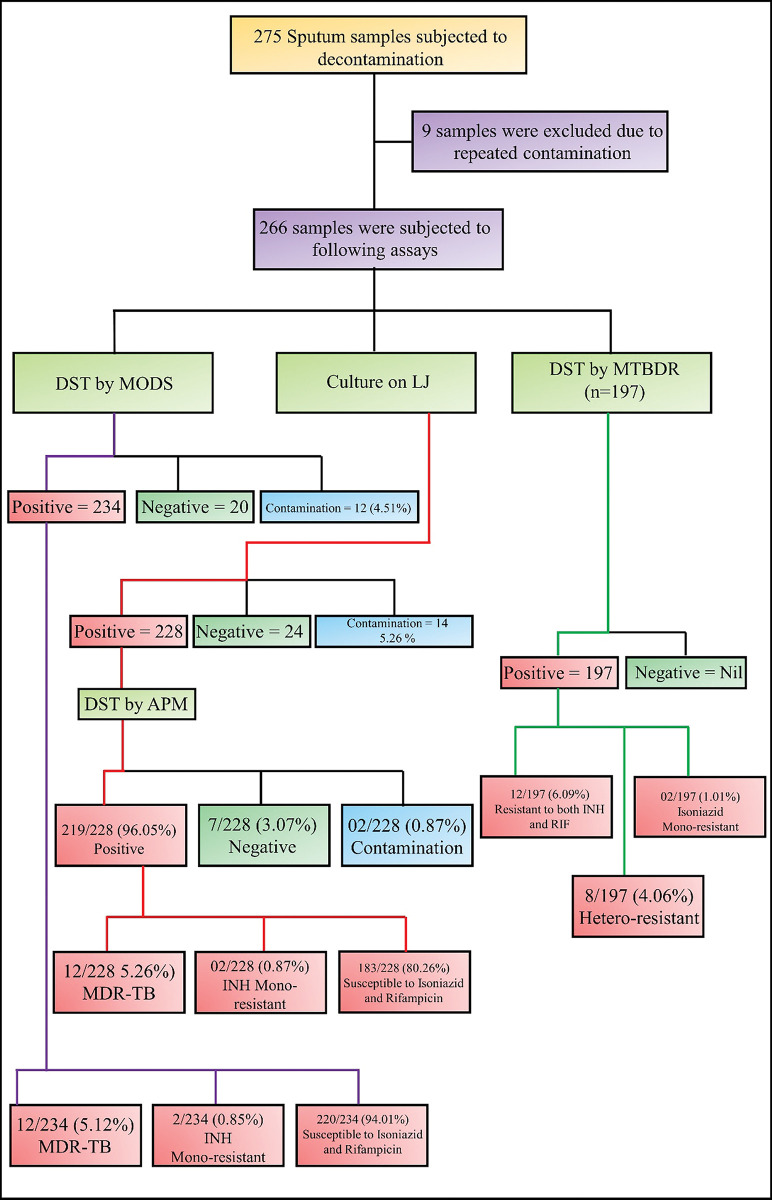
Flowchart of patient enrolment and analysis of results by different detection methods.

Out of 275 patients enrolled 9 (3.27%) were excluded as their samples showed contamination on repeated decontamination and the remaining 266 (96.72%) samples were subjected to culture on LJ medium, DST by Indirect Agar proportion method using Middlebrook 7H10 Agar and both culture and DST on modified MODS. Genotype MTBDR*plus* was performed on 197 (71.63%) samples. Fig 2 shows the results of these methods. The modified MODS assay was relatively easy to perform compared to the agar proportion method and MTBDR*plus* assay and did not require a highly skilled person for performing the assay and interpretation of the results.

Culture positivity rates were higher in modified MODS (87.96%) as compared to LJ (85.71%), but the difference observed was not statistically significant (p-value = 0.44). The performance of the modified MODS assay for the detection of tuberculosis in comparison to the reference gold standard i.e., the LJ method is shown in Table 2. Modified MODS showed substantial agreement with LJ (kappa value = 0.760) for the detection of tuberculosis.

**Table 2:** Sensitivity, specificity, positive predictive value and negative predictive value of DST by modified MODS for the detection of M. tuberculosis and INH or RIF resistance.

Modified MODS	Sensitivity	Specificity	PPV	NPV
**Detection of tuberculosis**	97.44% (94.50% - 99.05%)	80.00% (61.43% - 92.29%)	97.44% (94.89% - 98.73%)	80.00% (64.02% - 89.99%)
**Detection of INH-resistant isolates**	92.86	99.52	100	100
**Detection of RIF-resistant isolates**	100	100	100	100
**Detection of MDR isolates**	100	100	100	100

PPV: Positive predictive value

NPV: Negative predictive value

**Fig 3:**
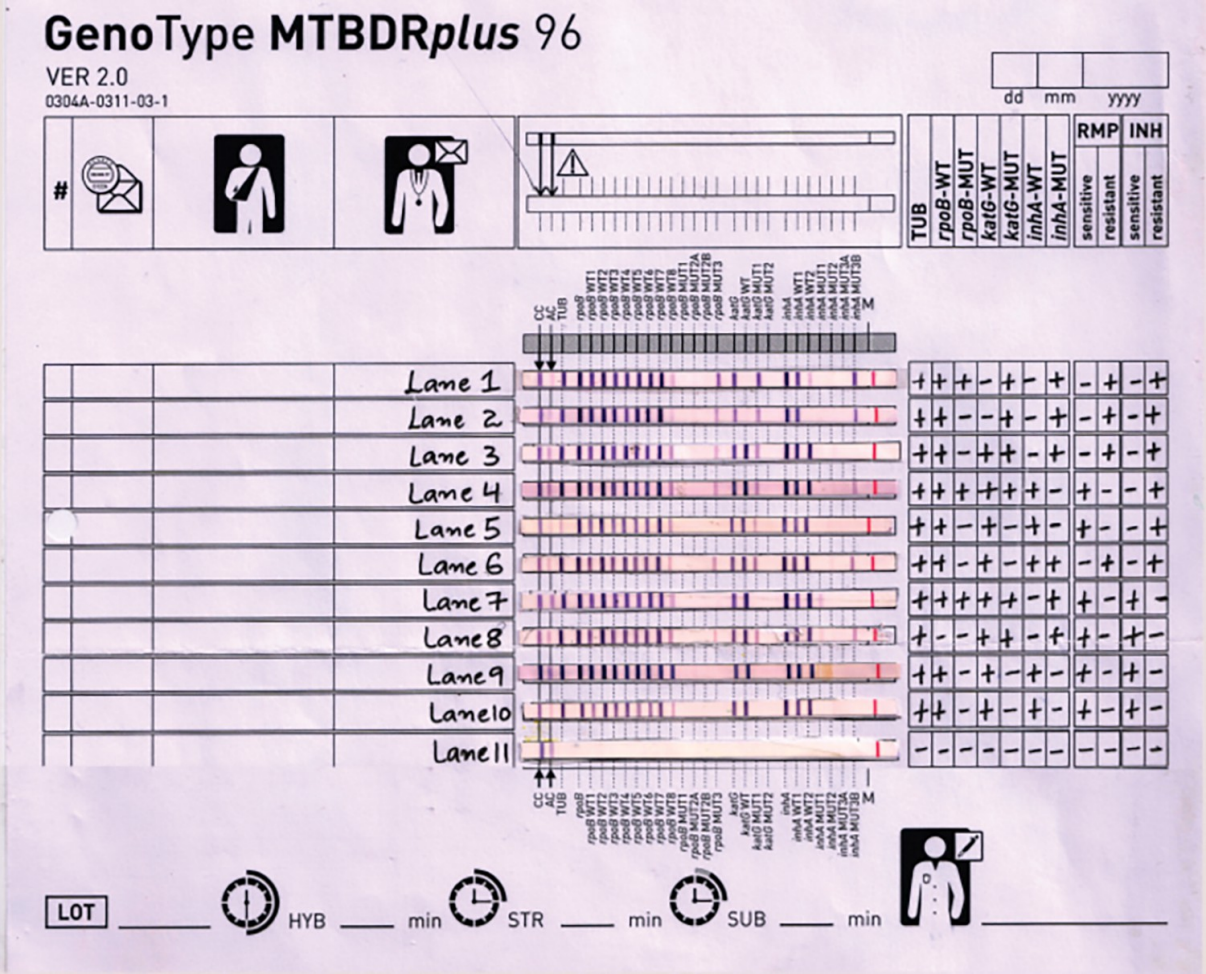
GenoType MTBDR-plus strip showing the mutational pattern of representative isolates (Lane 1) Multidrug-resistant tuberculosis, *rpoB* S531L mutation. (Lane 2) Multidrug-resistant tuberculosis, *rpoB* H526R mutation. (Lane 3) Multidrug-resistant tuberculosis, *katG* S315T2 mutation. (Lane 4) *M*. *tuberculosis*, INH monoresistant (*katG* S315T1 mutation). (Lane 5) *M*. *tuberculosis*, INH heteroresistant (mutation in *katG* 315 region). (Lane 6) Multidrug-resistant tuberculosis, *InhA* T8C mutation. (Lane 7) *M*. *tuberculosis*, INH heteroresistant (mutation in *InhA* -15 region). (Lane 8) *M*. *tuberculosis*, INH heteroresistant (mutation in *InhA* -16 region). (Lane 9) *Mycobacterium tuberculosis*, susceptible to isoniazid and rifampicin. (Lane 10) H37Rv Positive control. (Lane 11) Negative control (distilled water).

Drug susceptibility testing using modified MODS and agar proportion method showed the same detection rates for multidrug-resistant TB and Isoniazid mono-resistant however, susceptibility to isoniazid and rifampicin was better detected by modified MODS assay as compared to agar proportion method (Fig 2). The performance of the modified MODS assay in comparison to agar proportion method for drug susceptibility is given in table 2. A perfect agreement was seen between modified MODS and APM for the detection of isoniazid (kappa = 0.923) and rifampicin (kappa = 1) with a p-value = <0.001. GenoType MTBDR*plus* assay was done in 197 samples and the resistance patterns of these isolates are shown in (**Fig 2**). The mutational pattern which was observed is shown in Table 3 and Fig 3.

**Table 3:** The mutational pattern of drug-resistant *Mycobacterium Tuberculosis* strains.

Gene	Band	Gene Region/ Mutation	*rpoB* gene mutation band	Mutation	RIF Mono-resistant strains (n = 0)	INH Mono-resistant strains (n = 2)	INH Hetero-resistant Strains (n = 8)	MDR-TB strains (n = 12)
***rpoB***	WT 8	530–533	*rpoB* MUT 3	S531L	0	0	0	8 (66.66%)
	WT 7	526–529	-	H526R	0	0	0	4 (33.33%)
***katG***	*KatG* WT	315	*KatG* MUT2	S315T2	0	0	0	7 (58.33%)
	*KatG* WT	315	*KatG* MUT 1	S-315T1	0	1 (50.00%)	2 (25.00%)	0
	*KatG* WT	315	*KatG* MUT2	S-315T2	0	0	2 (25.00%)	0
***inhA***	*inhA* WT2	-8	*InhA* MUT 3A	T8C	0	0	0	5 (41.66%)
	*inhA* WT1	-15	*InhA* MUT 1	C-15T	0	1 (50.00%)	3 (37.50%)	0
	*inhA* WT1	-16	*InhA* MUT 2	A-16 G	0	0	1 (12.50%)	0

**Fig 4:**
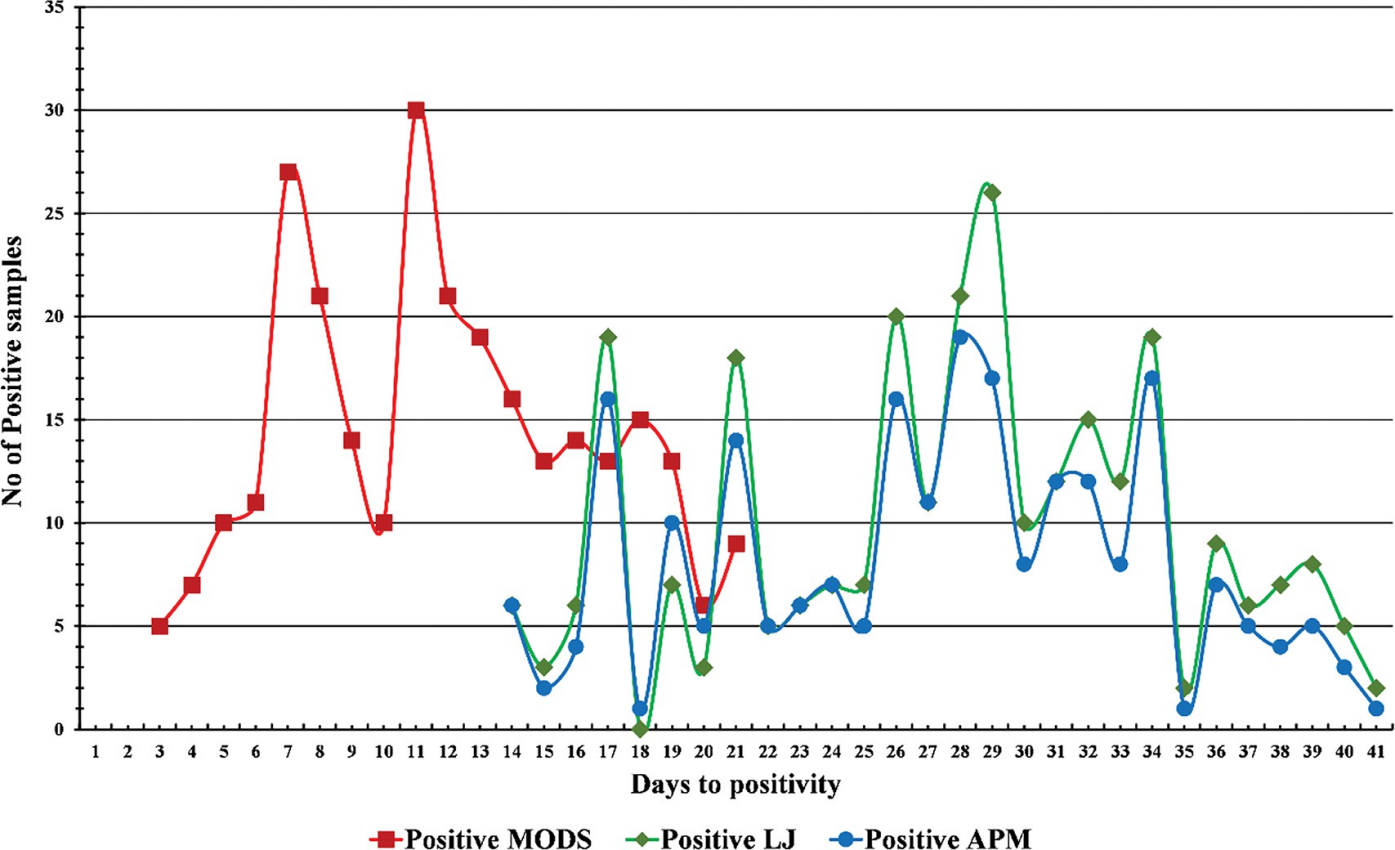
Time to positive culture for modified MODS, Lowenstein–Jensen (LJ) and APM.

The contamination rate was higher on LJ media as compared to modified MODS (Fig 2). The median time to sputum culture positivity was significantly shorter for modified MODS than for APM and LJ (9, 15 and 28 days, p < 0.001) (**Fig 4**).

**Fig 5:**
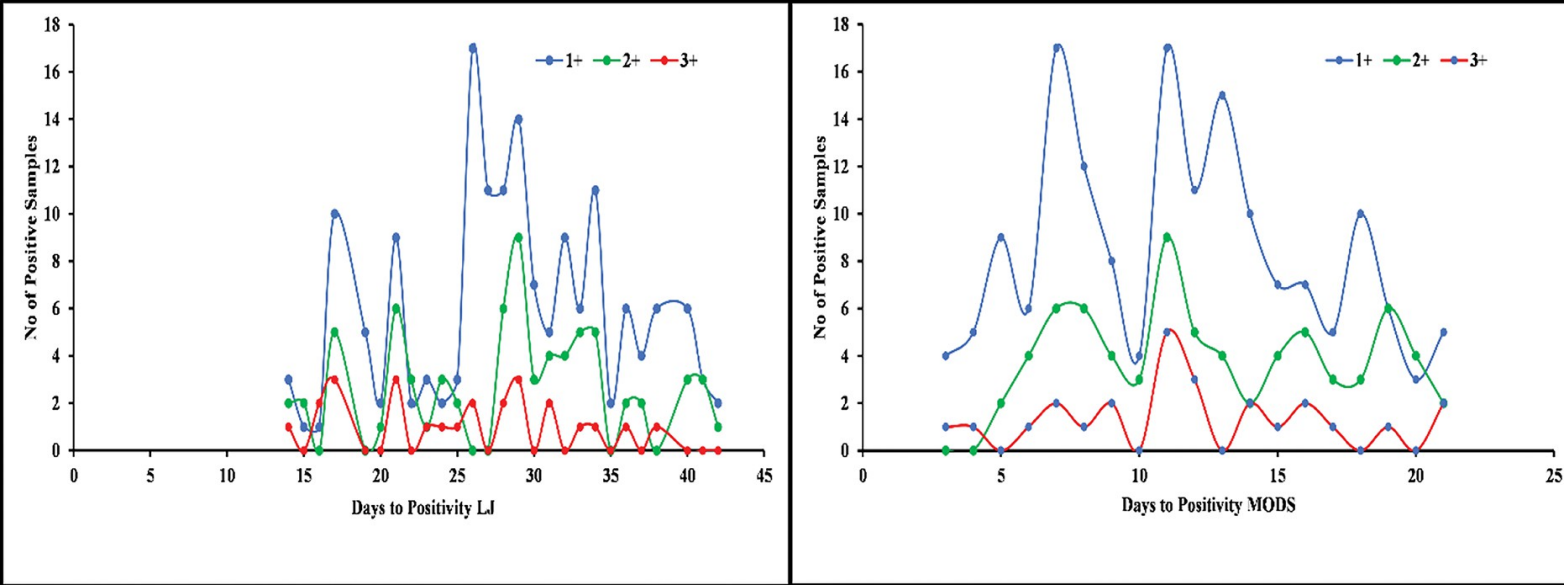
Time to LJ and modified MODS positivity in relation to smear grade.

For modified MODS and LJ, smear grading did not affect time to positivity (**Fig 5**). The mean TAT for the detection of *M*. *tuberculosis* ranged from 4–21 days for modified MODS and 16–21 days for APM. However, the TAT calculated for MTBDR plus was 24–48 hrs.

The cost for Conventional DST was 0.98$ (80 INR) which included the cost of LJ medium 0.31$ (25 INR) and APM media 0.67$ (55 INR). The cost calculated for modified MODS was 2.02 $ (165 INR). The cost of MTBDR*Plus* was 16.87 $ (1375 INR), but with use of negative and positive controls with each batch as per the manufacturer’s instructions increased the cost to Rs 1675 per test.

## Discussion

In the present study modified MODS was used for the rapid detection of tuberculosis and its drug susceptibility to isoniazid and rifampicin. The high level of concordance between the methods indicates that the drug susceptibility test results by the modified MODS assay and the traditional indirect agar proportion are reliable. Mutation pattern in a drug-resistant strain of tuberculosis has not been described previously from this region. GenoType MTBDR*plus* assay was performed in our study for the detection of INH and RIF resistance and the mutational pattern associated with the resistance.

The culture positivity rate was slightly higher in modified MODS than in the solid LJ culture however, the difference was not statistically significant. A similar observation was made in other studies [8,20]. The modified MODS had an overall good performance, with a sensitivity of 97.44% and specificity of 80%, and a substantial agreement (k = 0.77) with the indirect agar proportion method. In another study sensitivity of 89.0% and specificity of 92.3% was observed, however in this study, the results of MODS were compared with both LJ and Mycobacteria growth indicator tube method [21].

Resistance to isoniazid and rifampicin was detected in *M*. *tuberculosis*, which are the main drugs used in the short-course treatment of TB. The sensitivity and specificity for the detection of isoniazid-resistant isolates by modified MODS assay were 92.8% and 99.52% with PPV and NPV of 100% however, for the detection of rifampicin-resistant isolates sensitivity, specificity, PPV and NPV were 100%. Similar results were observed in other studies [20]. The performance for the detection of the rifampicin resistance by modified MODS assay was better as compared to the detection of isoniazid resistance with both showing perfect agreement i.e., isoniazid (kappa = 0.923) and rifampicin (kappa = 1). These findings are supported by another study that found excellent agreement (kappa = 0.932) rates for detecting isoniazid and rifampicin resistance using MODS assay [20,22]. Sensitivity, specificity, positive predictive value, and negative predictive value for the detection of multidrug-resistant TB by the modified MODS method was 100% in smear-positive patients. A concordance of 100% was observed for the detection of multidrug-resistant TB with a kappa value of 1 and had a perfect agreement, however, in some studies, 99% concordance has been reported [8]. In other studies, done for the diagnosis of multi-drug resistant tuberculosis lower sensitivity, specificity, positive predictive value, and negative predictive value were observed as compared to the findings of our study [8,20]. Brady et al., evaluated drug susceptibility in liquid culture and demonstrated the feasibility of MODS as a low-cost and effective tool in the early detection of multidrug-resistant TB. It also has a good concordance with phenotypic drug susceptibility results [19].

A low percentage of multidrug-resistant-TB and isoniazid monoresistance was seen in our study by both methods, with no mono-resistance to rifampicin. Resistance rates in our study were very low as compared to other studies conducted across India where monoresistance to rifampicin and isoniazid has been reported between 1–15%, 8.5–10% respectively and 20–55% being as multidrug-resistant [18].

GenoType MTBDR*plus* assay detected of multidrug-resistant resistances in 6.09%, isoniazid monoresistant in 1.01% and heteroresistance in 4.06% isolates. There was 98.68% concordance between modified MODS and agar proportion method assay for the detection of isoniazid resistances and 1.32% discordance however for the detection of rifampicin-resistant isolates there was 100% concordance between modified MODS and agar proportion method. Disagreements were reported for three isolates that were isoniazid sensitive by the modified MODS method but resistant by the agar proportion method assay. The results were verified by MTBDR*plus* assay which found these two samples to be sensitive and one sample to be heteroresistance.

In the case of isoniazid, there was discordance between the results of MTBDR*plus*, modified MODS and agar proportion method, as 8 (4.06%) isolates, heteroresistant by MTBDR*plus* were sensitive to isoniazid by modified MODS and agar proportion method. This discordance is believed due to the masked expression of phenotypic resistance in the agar proportion method in the presence of a predominantly sensitive bacterial population. Many studies have already demonstrated the feasibility of MTBDR*plus* assay as an effective tool in the early detection of multidrug-resistant tuberculosis and has good concordance with phenotypic drug susceptibility results [10,23–25]. In our study, 4.0% of isolates showed heteroresistance, which is lower than the 9.8% to 28.8% reported in other parts of India. The detection of hetero-resistant strains would ensure the timely initiation of multidrug-resistant- tuberculosis regimens in such patients. Patients harbouring such strains have better treatment outcomes compared to pure multidrug-resistant tuberculosis patients [26]. The low resistance in our study could be attributed to the fact that most of the patients included in the current study were new cases, the low incidence of HIV/AIDS in our community, and an effective tuberculosis control surveillance and monitoring programme.

The most prevalent mutation in multidrug-resistant TB was found in the mutational hot spot region of *rpoB* gene codon 531 with Ser531→Leu (66.66%) replacement in rifampicin-resistant strains. In line with our finding, this mutation (Ser531 → Leu) was detected among rifampicin-resistant strains varying from 47% to 70.5% in various studies [23,27–30]. The *katG* gene mutations account for the commonest mechanism of resistance for isoniazid. The most frequent mutation in our study was the S315T2 (58.3%). Mutation in the *InhA* gene was found to be 41.7% which was similar to the findings of the previous studies [23]. In contrast to our findings, other studies have reported low prevalence (5.4–21.1%) [23,27–31]. In isoniazid hetero-resistant strains mutation S315T1 and S315T2 in the *katG* gene were equally prevalent however, variations have been reported in studies [23,27,31,32]. The high prevalence of *katG* mutations contributes to most cases of isoniazid resistance in high-burden countries and leads to high-level isoniazid resistance [31,32]. In the *InhA* gene, the most prevalent mutation was C-15T (37.50%) followed by A-16 G (12.50%) mutation. Other studies have reported mutations of 41.7% in the *InhA* gene [23]. In isoniazid mono-resistant strains only two mutations were found S-315T1 (50%) in the *katG* gene and C-15T (50%) in the *inhA* gene. Other studies have reported a 42.9% mutation in the *InhA* gene for INH mono-resistant strains [23]

Turnaround time (TAT) for MTBDR*plus* was 1–2 workdays, 4–21 days for MODS and 10–21 days for agar proportion method assay. The median turnaround time of 9 days for MODS observed in the present study is in concordance with the other studies [20,21,33,34]. The contamination rate for both solid media and liquid media in the present study was within the accepted range of 3–5% for solid media and 3–7% for liquid media. Similar observations have been documented in other studies [20,21]. The cost of MTBDR*plus* was 10 times more than the Modified MODS and 21 times more than the agar proportion method assay including LJ media used for primary isolation, while modified MODS was twice as costly as the Indirect agar proportion method assay similar observations have been made in another study [35]. A study showed that the cost of MODS is lower than those of other available DST methods [36]. Cost is an important factor for any health intervention, particularly in resource-limited settings [37].Although the cost of MODS in the present study is high as compared to the Indirect agar proportion method it has the advantage of less turnaround time as both detection of TB and drug susceptibility can be performed simultaneously. The limitation of the study is that the GenoType MTBDR*plus* assay was performed on only 197 samples due to financial constraints.

## Conclusion

Modified MODS used in our study proved to be cost-effective, and user-friendly, with minimal risk to the handler and no cross-contamination between samples. Susceptibility could be performed directly from the sample by Modified MODS in contrast to the gold standard agar proportion method assay. The use of an inverted microscope with a screen instead of an objective made the viewing of bottles very easy and less tedious. Susceptible by Modified MODS was in perfect agreement with the gold standard method. More studies in different settings might be needed before the recommendation to put the test to routine use in mycobacteriology labs in low-income countries.
